# Screened prevalence of trichotillomania and its association with self-esteem among Saudi medical students: a cross-sectional study

**DOI:** 10.3389/fpsyt.2025.1673412

**Published:** 2025-10-24

**Authors:** Saleh A. Alghamdi

**Affiliations:** Psychiatry Department, College of Medicine, Imam Mohammad Ibn Saud Islamic University (IMSIU), Riyadh, Saudi Arabia

**Keywords:** Trichotillomania, medical students, prevalence, Saudi Arabia, hair pulling, body-focused repetitive behaviours, Rosenberg self-esteem scale, trichophagia

## Abstract

**Introduction:**

Trichotillomania is a chronic psychiatric syndrome characterized by an uncontrollable urge to pull out one’s hair. The current diagnostic criteria for Trichotillomania are as follows: hair pulling that leads to hair loss; attempts to reduce or stop hair pulling; significant distress or impairment; and the pulling cannot be attributed to another mental or physical condition.

**Aim:**

The major objective of the study was not to formulate an official clinical diagnosis but to get a preliminary understanding of the prevalence of Trichotillomania symptoms and their possible impact on self-esteem in the medical students in Saudi Arabia by using a self-reported checklist based on DSM-5 criteria.

**Subjects and methods:**

This cross-sectional study was conducted among medical students across various regions of Saudi Arabia. Data were collected through a self-administered online questionnaire designed to ensure broad accessibility and participation. The survey consisted of three main components: (1) sociodemographic information, including age, gender, marital status, and other relevant background variables; (2) a screening checklist based on the DSM-5 diagnostic criteria for Trichotillomania; and (3) the Rosenberg Self-Esteem Scale, used to assess participants’ levels of self-esteem.

**Results:**

The response rate was 439 (114%), and of the 439 medical students, 50.1% were females, and 64.2% were aged between 18 and 20 years. 39.4% had low self-esteem. The screened prevalence of Trichotillomania in this study was 11.4%. Trichotillomania was higher in Saudi nationals, those who had been married, those who had previously heard of Trichotillomania diagnosis, and those with low self-esteem. In multivariate regression analysis, having heard of Trichotillomania diagnosis and low self-esteem were identified as the significant independent risk factors for Trichotillomania.

**Conclusion:**

More than ten percent of medical students through this screening have shown symptoms of Trichotillomania. Symptoms of Trichotillomania potentially affect the mental conditions of students, including self-esteem. The screening has shown that the Trichotillomania was higher in females than males, but the difference was insignificant. Longitudinal studies are needed to extract more data about the Trichotillomania prevalence and its relative impact on mental health conditions.

## Introduction

Trichotillomania is a chronic psychiatric syndrome characterized by a continuous and intolerable need to pluck the hair out, most commonly from the scalp and eyebrows, leading to a noticeable loss of hair in affected areas (1). The disorder is usually accompanied by certain hair rituals, such as biting, chewing, swallowing, or playing with the hair (2). Literature indicates that 5% to 20% of the cases also have trichophagia, which is the compulsive eating of hair (3–5). Despite being discussed for more than a century in the medical literature (6), Trichotillomania was not recognized as a mental health disorder in the Diagnostic and Statistical Manual of Mental Disorders (DSM) by the American Psychiatric Association until the DSM-III-R (1987), when it was categorized as an impulse control disorder not otherwise classified. Trichotillomania was listed alongside obsessive-compulsive disorder (OCD), excoriation disorder, body dysmorphic disorder, and hoarding disorder in the chapter on obsessive-compulsive and associated disorders in the fifth edition of the DSM (DSM-5) (1). The following are the current diagnostic criteria for Trichotillomania: The diagnostic criteria include: hair pulling that results in hair loss, unsuccessful attempts to reduce or stop the hair pulling, significant distress or impairment caused by the behavior, and the hair pulling cannot be explained by another mental or physical condition (1). A scarcity of epidemiological studies remains; recent analyses estimate a point prevalence of trichotillomania at approximately 1%, with significantly elevated rates of hair-pulling symptoms in community samples, generally commencing in adolescence or early adulthood (7–9). The average age for the onset of symptoms is 13 years old. The age curve of onset of symptoms is bimodal, with the first peak in childhood and a second in adolescence (7–9). The findings about the sex ratio are discordant, with the sex ratio ranging from two females to one male and seven females to one male (1). At present, the MGH-HPS (Massachusetts General Hospital Hair Pulling Scale) is a validated scale that is used to evaluate the severity and impairment caused by hair pulling (10). A study conducted by Magdalena Grzesiak et al. assessed the prevalence and comorbidity of Trichotillomania in young adults, revealing that 62.5% of affected individuals had anxiety disorders—diagnosed at a higher rate than both the general population and those without Trichotillomania (20.2%)—and additionally indicated that the condition is often associated with nail biting or skin picking (11). In 2020, Andre P. Bezerra conducted a study in the UK to evaluate the prevalence, correlation, and independent impact of Trichotillomania on quality of life (QoL). Participants with probable Trichotillomania were more likely to have co-occurring probable alcohol use disorder, tobacco use disorder, and depression. Suicidal ideation and childhood sexual abuse were independently associated with probable Trichotillomania (ORadj = 1.917; 95% CI: 1.224-3.003) and probable Trichotillomania (ORadj = 1.221; 95% CI: 1.098-1.358), respectively. A positive Trichotillomania screen was also associated with worse physical and mental quality of life (12).

Trichotillomania is a rare psychiatric disorder, and there is a paucity of studies on this condition within Arab Middle Eastern communities, particularly in Saudi Arabia (13, 14). Bibliometric analyses substantiate this disparity, revealing that research activity on Trichotillomania is predominantly concentrated in the United States, Turkey, and Germany (15). However, despite the widespread recognition of structured clinical interviews as the gold standard for diagnosing Trichotillomania they were not a viable option for this study due to a variety of practical considerations. The in-person assessments were challenging to administer due to the limited resources and the dispersion of medical students throughout Saudi Arabia. A self-reported assessment that adhered to DSM-5 standards was implemented as an alternative. This method simplified the process of collecting data from a large sample in a manner that adhered to standard diagnostic guidelines.

The primary objective of the study was not to establish official clinical diagnoses but rather to gain an initial understanding of the prevalence of Trichotillomania symptoms and their potential correlation with self-esteem among medical students. Additionally, we aimed to make participation as smooth and accessible as possible. An anonymous online survey, characterized by simplicity, is more likely to enhance engagement and promote truthful responses due to the academic pressures faced by medical students. While acknowledging that self-report measures may inflate prevalence rates without clinical validation, this method offers important preliminary insights and recommends further diagnostic research and targeted mental health support within academic settings.

## Methods

### Study design

This study was conducted in Saudi Arabia between February 2023 and February 2024 using an observational cross-sectional design. Its primary objective was to determine the prevalence of Trichotillomania symptoms among medical students (without establishing a clinical diagnosis) and to investigate their potential impact on self-esteem. The survey was designed to be administered to students from various regions of the country and was divided into three sections. The initial section gathered data regarding the individual’s age, gender, country, place of domicile, university, academic year, and any history of mental illness. The second section used a checklist based on DSM-5 criteria to screen for Trichotillomania symptoms, and participants who endorsed all five DSM-5 criteria items were classified as screening positive for Trichotillomania. The DSM-5-based checklist was developed for this study based on the established diagnostic criteria (1). The third section assessed self-esteem using the Rosenberg Self-Esteem Scale (16).

#### Sampling size and statistical method

To ensure adequate statistical power, we determined the required sample size with the Raosoft calculator (target n = 385; margin of error ±3.3% for the observed prevalence) and enrolled 439 participants, surpassing this threshold. Participants were recruited through convenience sampling. Data were analyzed using SPSS, employing descriptive statistics, t-tests for continuous variables, and chi-square (χ²) tests for categorical variables.

### Ethical consideration

Based on the Declaration of Helsinki, the Institutional Review Board approval was obtained from the Medical Research Unit, Faculty of Medicine, Imam Mohammad ibn Saud Islamic University, Riyadh, Saudi Arabia (IRB Number: 435/22-2-2023). Involvement in this study is entirely voluntary; all participants are informed and asked to provide their consent before joining. Participation does not result in any financial compensation.

### Statistical analysis

**Figure 1:**
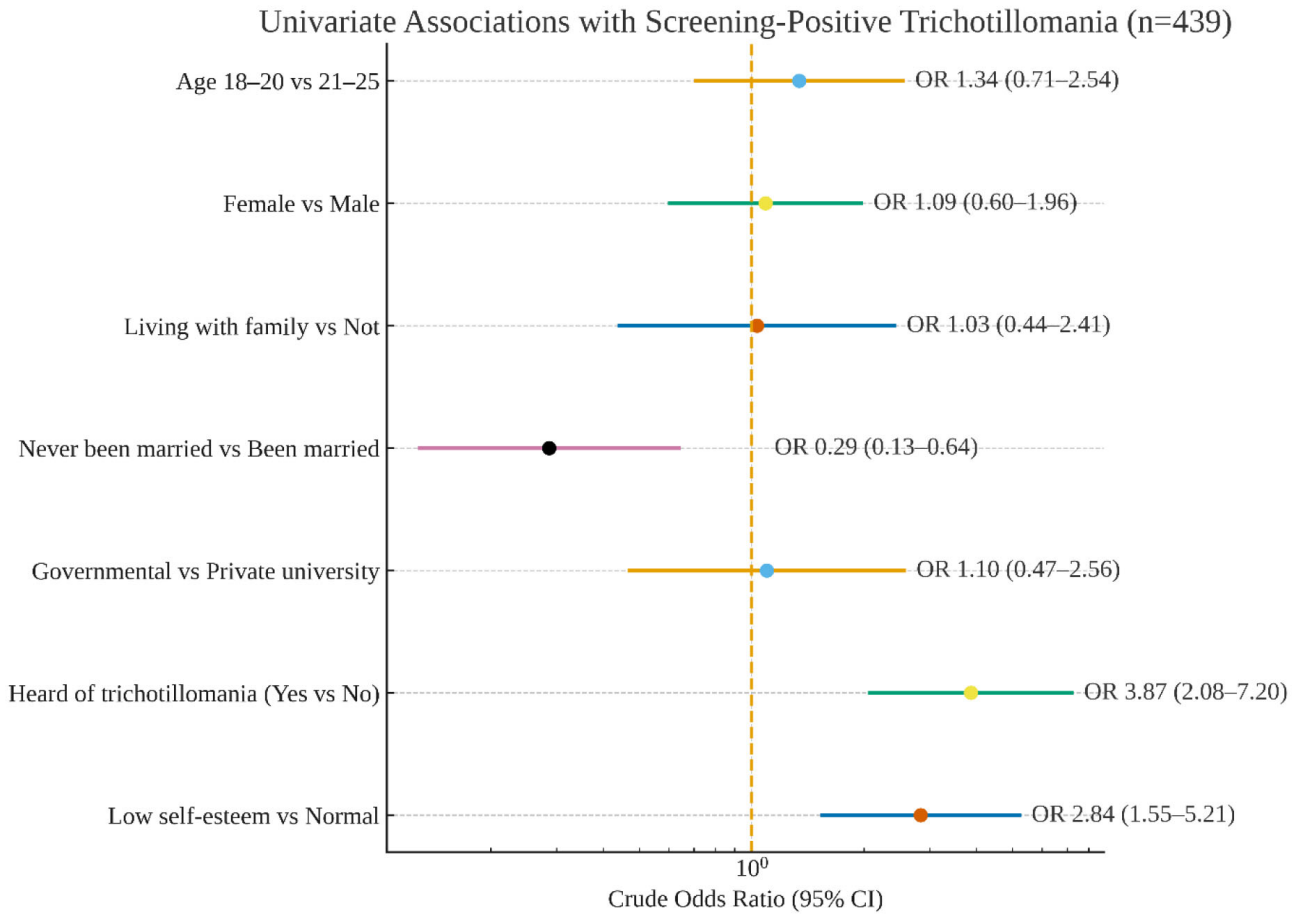
Forest plot of multivariable logistic regression for screening-positive trichotillomania (n = 439). N.B. The forest plot was generated using R statistical software (latest stable version at the time of analysis; R Foundation for Statistical Computing, Vienna, Austria).

Categorical variables were presented using numbers and percentages. Continuous variables were given as means and standard deviations. Univariate analysis was performed to determine the factors that influence Trichotillomania. Significant results were gathered in a multivariate regression model to determine the significant independent risk factors of trichotillomania, with corresponding odds ratios as well as 95% confidence intervals. A p-value of <0.05 was used to indicate statistical significance. All data analyses were performed using the statistical package for social sciences, version 26 (SPSS, Armonk, NY: IBM Corp.). The multivariable logistic regression results were represented in a forest plot (**Figure 1**), which depicted adjusted odds ratios (aORs) with 95% confidence intervals on a logarithmic scale.

## Results

This study enrolled four hundred thirty-nine medical students. **Table 1** presents the socio-demographic characteristics of the medical students. 64.2% were between 18 and 20 years old. Females (50.1%) were slightly higher than males (49.9%). Saudi nationality constitutes most of the students (92.9%). Students who lived in the Central Region constitute 45.3%. A great proportion of students lived with their families. Nearly all were single (91.8%). Students who were in the first-year levels constituted 32.3%. The most common type of university was governmental (85%). In addition, 37.1% had previously heard of Trichotillomania.

**Table 1:** Socio-demographic characteristics of the medical students (n=439).

Study variables	N (%)
Age group	
• 18–20 years	282 (64.2%)
• 21–25 years	157 (35.8%)
Gender	
• Male	219 (49.9%)
• Female	220 (50.1%)
Nationality	
• Saudi	408 (92.9%)
• Non-Saudi	31 (07.1%)
Region of residence	
• Central Region	199 (45.3%)
• Southern Region	73 (16.6%)
• Northern Region	56 (12.8%)
• Eastern Region	35 (08.0%)
• Western Region	76 (17.3%)
With whom do you live?	
• With family	376 (85.6%)
• With friends	19 (04.3%)
• Alone	44 (10.0%)
Marital status	
• Single	403 (91.8%)
• Married	27 (06.2%)
• Divorced	09 (02.1%)
Academic year level	
• First Year	142 (32.3%)
• Second Year	130 (29.6%)
• Third Year	87 (19.8%)
• Fourth Year	40 (09.1%)
• Fifth Year	20 (04.6%)
• Sixth Year	20 (04.6%)
Type of university	
• Governmental	373 (85.0%)
• Private	66 (15.0%)
Have you heard before about the diagnosis of Trichotillomania?	
• Yes	163 (37.1%)
• No	276 (62.9%)

Regarding the screening of Trichotillomania (**Table 2**), 48.1% indicated recurrent pulling out of hair, resulting in hair loss; 45.3% had the attitude of repeated attempts to decrease or stop hair pulling, while 40.8% disclosed that hair pulling causes clinically significant distress. Accordingly, the screened prevalence of Trichotillomania in this study was 11.4%, and the rest were negative (88.6%). Regarding the self-esteem questionnaire, the top three statements that showed the highest ratings were “On the whole, I am satisfied with myself” (mean score: 2.10), followed by “I feel I do not have too much to be proud of” (mean score: 1.69), and “At times, I think I am no good at all” (mean score: 1.68). The total mean self-esteem score was 15 (SD 2.67), with low self-esteem constituting 39.4%, and the rest were normal (60.6%).

**Table 2:** Screening of Trichotillomania symptoms and its impact on self-esteem (n=439).

Trichotillomania items	Yes (%)
1. Recurrent pulling out of your hair, resulting in hair loss.	221 (48.1%)
2. Repeated attempts to decrease or stop hair pulling.	199 (45.3%)
3. The hair pulling causes clinically significant distress or impairment in social, occupational, or other important areas of functioning.	179 (40.8%)
4. The hair pulling or hair loss is not attributable to another medical condition (e.g., a dermatological condition).	161 (36.7%)
5. The hair pulling is not better explained by the symptoms of another mental disorder (e.g., attempts to improve a perceived defect or flaw in appearance in body dysmorphic disorder).	164 (37.4%)
Screened Prevalence of Trichotillomania	
• Positive	50 (11.4%)
• Negative	389 (88.6%)
Self-esteem items	Mean ± SD
1. On the whole, I am satisfied with myself	2.10 ± 0.83
2. At times, I think I am no good at all.	1.68 ± 1.01
3. I feel that I have a number of good qualities. *	0.73 ± 0.79
4. I am able to do things as well as most other people.	1.63 ± 0.48
5. I feel I do not have much to be proud of. *	1.69 ± 1.04
6. I certainly feel useless at times	1.54 ± 1.05
7. I feel that I’m a person of worth, at least on an equal plane with others.	1.34 ± 1.02
8. I wish I could have more respect for myself. *	1.07 ± 1.00
9. All in all, I am inclined to feel that I am a failure. *	1.67 ± 1.03
10. I take a positive attitude toward myself. *	0.95 ± 0.90
Total self-esteem score	15.0 ± 2.67
Level of self-esteem	N (%)
• Low (Score <15)	173 (39.4%)
• Normal (Score ≥15)	266 (60.6%)

Response to self-esteem items has a category range from “strongly disagree” coded with 0 to “strongly agree” coded with 3.

* Reversed coded item.

In univariate analysis (**Table 3**), it was revealed that those who had been married (p=0.001), those who had previously heard of Trichotillomania (p<0.001), and those with low self-esteem were more likely to develop Trichotillomania. Nationality was excluded from the multivariate analysis because of homogeneity, as all participants with Trichotillomania were Saudi.

**Table 3:** Univariate analysis of the factor that influences Trichotillomania (n=439).

Factor	Prevalence of Trichotillomania		P-value ^a^
	Positive (n=50)	Negative (n=389)	
Age group			
• 18–20 years	35 (70.0%)	247 (63.5%)	0.366
• 21–25 years	15 (30.0%)	142 (36.5%)	
Gender			
• Male	24 (48.0%)	195 (50.1%)	0.777
• Female	26 (52.0%)	194 (49.9%)	
With whom do you live?			
• Living with family	43 (86.0%)	333 (85.6%)	0.940
• Not living with family	07 (14.0%)	56 (14.4%)	
Marital status			
• Never been married	40 (80.0%)	363 (93.3%)	0.001 **
• Been married	10 (20.0%)	26 (06.7%)	
Type of university			
• Governmental	43 (86.0%)	330 (84.8%)	0.828
• Private	07 (14.0%)	59 (15.2%)	
Have you heard before about the diagnosis of Trichotillomania?			
• Yes	33 (66.0%)	130 (33.4%)	<0.001 **
• No	17 (34.0%)	259 (66.6%)	
Level of self-esteem			
• Low	31 (62.0%)	142 (36.5%)	0.001 **
• Normal	19 (38.0%)	247 (63.5%)	

^a^P-value (The probability) has been calculated using Chi-square test.

** Significant at p<0.05 level.

When conducting a multivariate regression analysis (**Table 4**), it was found that medical students who have heard of Trichotillomania were predicted to increase the risk of developing Trichotillomania by at least 3.44 times (AOR = 3.444; 95% CI = 1.813–6.544; p<0.001). Compared to students with normal self-esteem, students who had low self-esteem were predicted to increase the risk of developing Trichotillomania by at least 2.59-fold (AOR = 2.593; 95% CI = 1.384–4.857; p=0.003). No significant difference was observed between the developing Trichotillomania and marital status after adjustment to a regression model (p=0.085). The multivariable logistic regression is summarized in **Figure 1**. Specifically, prior awareness of trichotillomania was independently associated with screening positive (aOR=3.444; 95% CI = 1.813–6.544; p<0.001), low self-esteem showed a similar association (aOR=2.593; 95% CI = 1.384–4.857; p=0.003), and marital status (never vs. been married) was not significant after adjustment (aOR=0.473; 95% CI = 0.201–1.109; p=0.085).

**Table 4:** Multivariate regression analysis to determine the significant independent risk factors associated with Trichotillomania (n=439).

Factor	AOR^a^	95% CI^b^	P-value^c^
Marital status			
• Never been married	0.473	0.201 – 1.109	0.085
• Been married	Ref		
Have you heard before about the diagnosis of Trichotillomania?			
• Yes	3.444	1.813 – 6.544	<0.001 **
• No	Ref		
Level of self-esteem			
• Low	2.593	1.384 – 4.857	0.003 **
• Normal	Ref		

^a^AOR: Adjusted Odds Ratio.

^b^CI : Confidence Interval.

^c^P-value (The probability) has been calculated using Chi-square test.

** Significant at p<0.05 level.

## Discussion

This study is carried out to determine the screened prevalence of Trichotillomania and evaluate its association with self-esteem by using a self-reported checklist based on DSM-5 criteria. According to the DSM-5 self-reported checklist, there was a high prevalence of Trichotillomania among medical students. Our screened prevalence of 11.4% aligns with recent student and regional samples and surpasses the majority of general-population estimates documented in contemporary studies (7, 8, 13, 14, 17). A recent meta-analysis indicated that the community prevalence of hair-pulling behaviours was around 8–9%, but the prevalence of diagnostic-threshold trichotillomania was lower, offering valuable context for our screening-based estimate (7). The higher rate of screened Trichotillomania among medical students was quite alarming. University institutions should devise an intervention program to address the growing prevalence of this disease. This condition could have severe health consequences if not addressed. Thus, efforts to minimize this burden are imperative. Data from our study suggest that an association between Trichotillomania symptoms, low self-esteem, and influencing mental conditions was observed. In this study, approximately 62% of students with Trichotillomania have concurrent low self-esteem, and this difference was statistically significant (p=0.001). After conducting a predictive model, students with low self-esteem were more than twice as likely to be suffering from Trichotillomania. This outcome is in agreement with the study published in Canada (18). Recent studies indicate that hair-pulling symptoms correlate with poorer mental health outcomes and increased impairment (8, 12, 19, 20). Supporting these accounts, Grant et al. disclosed that respondents with Trichotillomania and co-occurring anxiety exhibited worse symptoms of hair-pulling disorder (HPD) (21). They tended to have co-occurring depression and a family history of OCD (22). Incidentally, a previous report done in South Africa (23) suggests that obsessive-compulsive disorder (OCD) and Trichotillomania symptoms could worsen comparatively during the menstrual period; however, OCD worsening was more likely related to pregnancy or puerperium. Previous information on Trichotillomania diagnosis increased the risk of Trichotillomania by at least 3.44 times, which could be related to the awareness of Trichotillomania that may correlate with symptom recognition or help-seeking behaviour rather than causation (15). This did not seem to agree with the study of Mikhael et al. (17). Medical students who were aware of psychiatric conditions demonstrated better knowledge about Trichotillomania; however, they were less likely to be affected by this condition. Similarly, according to the study by Subki et al. (13), a history of hair-pulling disorder was seen in many subjects, but the probability of Trichotillomania was relatively less (<2%) (15). Not opposing these reports, Özten et al. (24) revealed that the correlation between the severity of post-traumatic stress symptoms and self-harming behavior yielded insignificant results and this led them to speculate that the developing Trichotillomania or skin-picking symptoms guide the patients to cope with invasive thoughts attributed to trauma (25).

A study suggested that hair-pulling disorders (HPD) were more prevalent in unmarried, unemployed, and not living with family (14). In contrast, a previous report conducted in the USA (8) documented insignificant associations between Trichotillomania and socio-demographic variables such as age, gender, education, monthly income, and racial-ethnic background. In our study, however, the prevalence of Trichotillomania was statistically significantly higher in Saudi nationals and those who had been married, but it was insignificant in terms of age, gender, living with family, and the type of university. Hence, attention should be focused on the groups showing increased Trichotillomania prevalence, as they may exhibit the worst-case scenario in terms of physical and mental functions.

Notably, nearly forty percent of the students had low self-esteem, which could be due to the burden of Trichotillomania. Immediate psychological intervention should be in place, as this burden may negatively affect the student’s academic performance. Given these reports, evidence across publications implicated that Trichotillomania was associated with multiple psychological comorbid conditions. For instance, Grzesiak et al. (9) indicated that young adults in Poland with Trichotillomania had overlapping psychological disorders such as anxiety and OCD (12). Houghton et al. (19) documented that individuals with HPD (38.8%) had another current psychiatric diagnosis, and nearly eight percent had another lifetime psychiatric diagnosis affecting quality of life (19). Likewise, Gerstenblith et al. (20) documented that Trichotillomania had excess comorbidity with several conditions from various DSM-IV criteria, such as tic disorders, mood disorders, alcohol dependence, impulse-control disorders, anxiety disorders, and bulimia nervosa. However, co-occurrence strengths were highest for kleptomania, pyromania, and OCD (20).

### Future research perspectives

More than cross-sectional, screening-only strategies are needed to determine diagnostic prevalence and incidence in Saudi universities. Probability-based, multicentre cohorts should adopt a two-stage approach (Arabic self-report screener followed by clinician-administered structured interviews) and psychometric validation of all instruments in the MGH-HPS and self-esteem scales. Multilevel/SEM longitudinal studies are needed to record symptom trajectories, test mechanisms (e.g., whether self-esteem mediates relationships between academic stress and hair-pulling), and evaluate modifiers (sex, academic stage, area). Standardized comorbidity and stress batteries and qualitative interviews should clarify burden, stigma, and help-seeking barriers. Beyond description, pragmatic campus trials (psychoeducation, habit-reversal, CBT/mindfulness) should report feasibility, acceptability, and symptoms and quality of life impacts. Preregistration, STROBE/CONSORT, sampling weights, and de-identified data and code improve rigor and comparability.

## Conclusion

Trichotillomania symptoms were detected in approximately one in ten medical students, and a substantial association was observed between these symptoms and low self-esteem. These results demonstrate the prevalence of Trichotillomania among medical students and underscore the necessity of promoting awareness and offering targeted mental health care. However, it is important to consider the following limitations when evaluating these results. A self-reported DSM-5 checklist can be employed for large-scale screening; however, it does not serve as a substitute for a clinical diagnosis and may have resulted in overestimations of prevalence due to potential false positives. Additionally, the study’s cross-sectional nature renders it impossible to conclude that symptoms of Trichotillomania are precipitated by self-esteem. The study’s convenience sampling and lack of clinical follow-up for those who tested positive may make the results less accurate or useful for diagnosing others.

## Data Availability

The raw data supporting the conclusions of this article will be made available by the authors, without undue reservation.
